# A Conservative Approach to Ceramic Laminates in the Anterior Region: A Clinical Report

**DOI:** 10.7759/cureus.68137

**Published:** 2024-08-29

**Authors:** Laura S Osorio-Vélez, Juan D Serna-Ceballos, Federico Latorre, Carlos M Ardila

**Affiliations:** 1 Prosthodontics Postgraduate Program, Faculty of Dentistry, Universidad de Antioquia, Medellín, COL; 2 Biomedical Stomatology Research Group, Faculty of Dentistry, Universidad de Antioquia, Medellín, COL

**Keywords:** aesthetic dentistry, ceramic veneers, digital dentistry, cad-cam, dental restoration

## Abstract

This clinical report presents a conservative approach to restoring aesthetic function in anterior maxillary teeth using digital dental technologies. A 40-year-old female patient sought treatment to enhance the aesthetics of her anterior maxillary teeth. The clinical examination revealed wear on the incisal surfaces and gingival asymmetry. Utilizing a digital workflow, intraoral and extraoral data were acquired through clinical photographs and an intraoral scanner. The patient's restorative needs were assessed using the 3Shape Smile Design system (3Shape, Copenhagen, Denmark). Minimal-thickness ceramic restorations were designed and fabricated using Computer-Aided Design-Computer-Aided Manufacturing (CAD-CAM) technologies. Lithium disilicate was selected for its strong mechanical properties and adhesive capabilities. The restorations were then placed using an adhesive cementation protocol under magnification. Digital technologies facilitated precise diagnosis, planning, and execution of the treatment. The ceramic restorations provided excellent aesthetic outcomes, aligning with the patient's expectations. The minimal thickness of the restorations ensured the preservation of natural tooth structure while maintaining structural integrity and adhesion performance. This case underscores the advantages of a digital workflow in achieving optimal restorative outcomes. The digital dental workflow demonstrated in this case report provides a reliable, efficient, and patient-centric approach to minimal thickness restorations, highlighting the potential for future advancements in restorative dentistry.

## Introduction

Modern oral rehabilitation procedures have been made simpler and more efficient using clinical magnification, intraoral scanning, and adhesive materials. These advances not only help to preserve dental structures but also provide guidance for dental professionals, leading to improved quality of care. A comprehensive evaluation of the patient, including clinical photography, intraoral scanning, and magnification with a dental microscope, offers more accurate information for better treatment planning [1,2]. The use of thin and fine-grain burs, careful handling of soft tissues with small thickness retraction cords, and minimal dental preparation results in thin diameter restorations that better protect the periodontium and provide superior adhesion performance. This requires rethinking treatment protocols, and using images and augmented vision to ensure reliable and predictable practice [3,4].

There is a growing demand for aesthetic dental procedures, with ceramic laminates becoming a popular choice due to their durability and natural appearance [1,3]. Despite the advancements in materials and techniques, there remains a need for detailed protocols that minimize invasiveness while maximizing aesthetic outcomes. This report aims to fill this gap by presenting a detailed protocol for the restoration of ceramic laminates (e.max Computer-Aided Design (CAD); Ivoclar Vivadent, Liechtenstein) on patient teeth with minimal thickness (0.4 mm), incorporating various digital tools and technologies to assist clinicians in enhancing their treatment methods.

The case involved restoring the right maxillary canine, right maxillary central incisor and left maxillary central incisor of a woman who expressed dissatisfaction with the worn and fractured incisal edges of her teeth. The specific objectives of this report are to demonstrate the clinical steps involved, highlight the importance of minimal thickness restorations, and emphasize the significance of avoiding excessive treatment to achieve patient satisfaction. The report concludes by underscoring the importance of utilizing minimally invasive restorations to achieve optimal patient outcomes.

## Case presentation

A 40-year-old female patient, with no prior medical or dental history and no previous treatments in the affected area, visited the University of Antioquia Faculty of Dentistry, Medellín, Colombia, for a consultation to improve the aesthetics of her anterior maxillary teeth. During the clinical examination using a microscope (Omni Pico, Zeiss, Oberkochen, Germany), wear on the incisal surfaces of the right maxillary canine, right maxillary central incisor, and left maxillary central incisor was observed, resulting in a straight smile that did not align with the lower lip. There was also a 1 mm discrepancy in the gingival margins between the right and left maxillary central incisors, causing asymmetry.

To analyze and plan the case, the digital patient protocol by Vandenberghe was implemented, which involves acquiring digitized patient data through extraoral and intraoral clinical photographs after an intraoral capture (TRIOS 3, 3Shape, Copenhagen, Denmark) [2]. The clinical photographic protocol was conducted using a Single Lens Reflex (SLR) camera (T6i, Canon, Tokyo, Japan) and a 100 mm macro lens (Canon), with appropriate lighting provided by a ring flash (Canon) and a strobe flashlight (Neewer, Shenzhen, China). The protocol involved taking extraoral and dentolabial relationship photographs.

**Figure 1:**
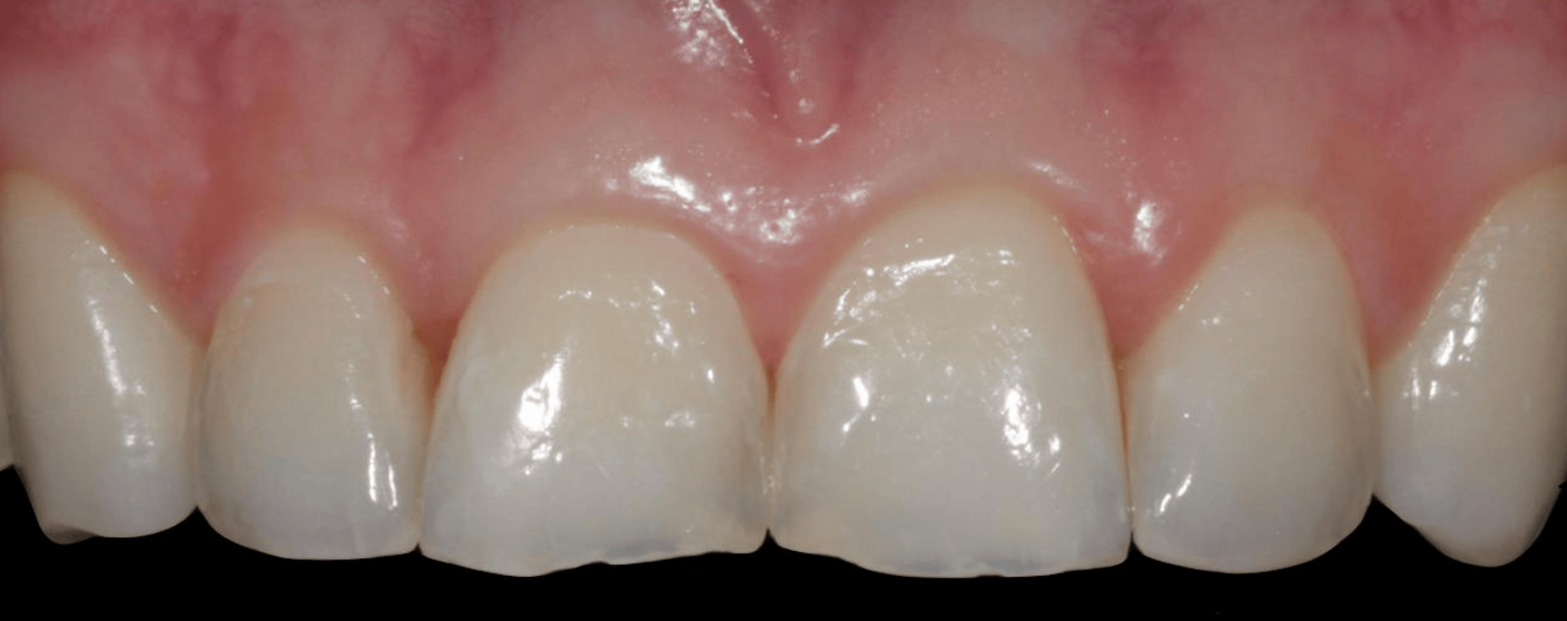
Frontal view of the maxillary anterior dentition demonstrating asymmetry of the central incisors.

Intraoral photographs were used for dental analysis and included images of the teeth in occlusion, the upper and lower arches, and both sides of the teeth (Figure 1).

**Figure 2:**
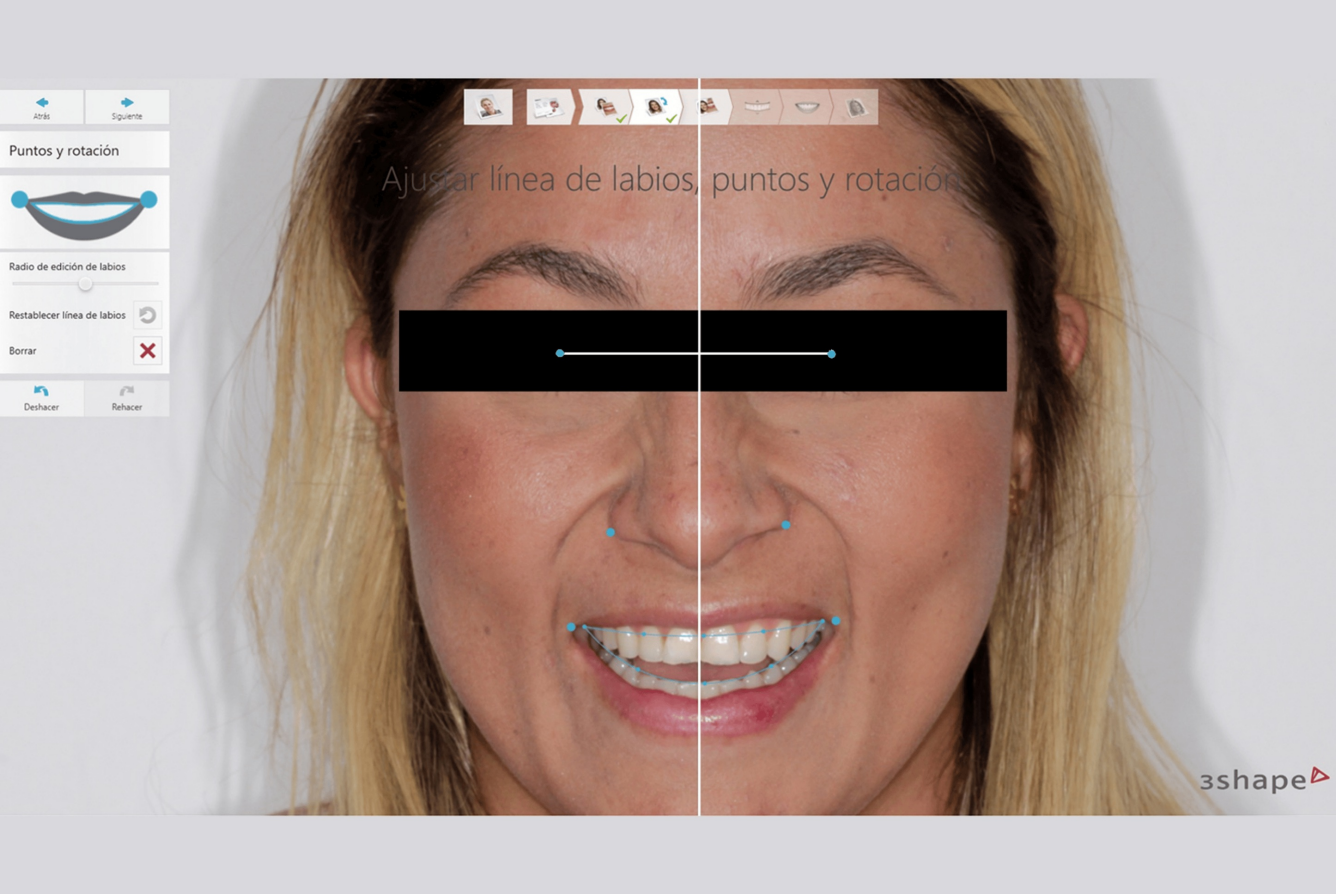
Extraoral virtual treatment planning using 3Shape Smile Design software

A digital dentogingival analysis was then performed using the 3Shape Smile Design system (3Shape, Copenhagen, Denmark) to identify the patient's restorative needs and emulate their tooth structure (Figure 2).

**Figure 3:**
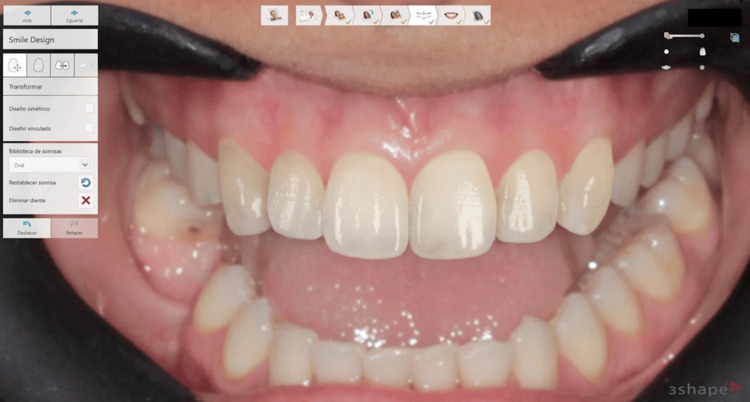
Virtual simulation of the proposed treatment outcome

The analysis showed that the patient needed a ceramic fragment restoration for tooth 6 and ceramic laminate veneers with an average thickness of 0.4 mm for teeth 8 and 9. Additionally, the possibility of improving gingival symmetry by leveling the gingival margin of tooth 8 with that of tooth 9 was identified (Figure 3).

After discussing the treatment plan with the patient, she agreed with most of the virtual restoration results. The patient signed a consent form and agreed to have photographic records used for academic publications but declined the periodontal procedure for gingival leveling.

The following steps were taken: a 3D model was printed from the digital wax-up using Formlabs (Formlabs, Somerville, USA). The resulting diagnostic wax-up was used to create a transparent silicone matrix with Memosil (Kulzer, Hanau, Germany), which was then filled with a bis-acrylic color A1 material (Luxatemp, DMG, Hamburg, Germany). This was applied to the patient's mouth as a mock-up, allowing her to experience both the functional and aesthetic results. After evaluating the mock-up, the patient accepted the treatment and proceeded with the final restorations.

The final material should have properties like enamel, as it is the structure being replaced. When selecting materials for minimal-thickness restorations, it is important to choose a material that allows for adhesive cementation [5]. Lithium disilicate was chosen for its strong mechanical properties, good adhesion, and predictable performance [6].

To prepare for the restoration, round yellow grain diamond burs were used to control the depth of the ceramic restorations with minimum thickness. This was done using a mock-up in the mouth to preserve as much enamel as possible for the adhesive technique [7]. Afterwards, thin tapered yellow diamond burs (with diameters of 0.12 mm and 0.10 mm) were used to reduce aggressive wear. The restoration was finished with Sof-Lex discs (3M, St. Paul, USA). Before dental preparation, a 000-knitted retraction cord (Zhermack, Badia Polesine, Italy) was placed in the cervical area to gently displace the tissues and provide better visual access.

**Figure 4:**
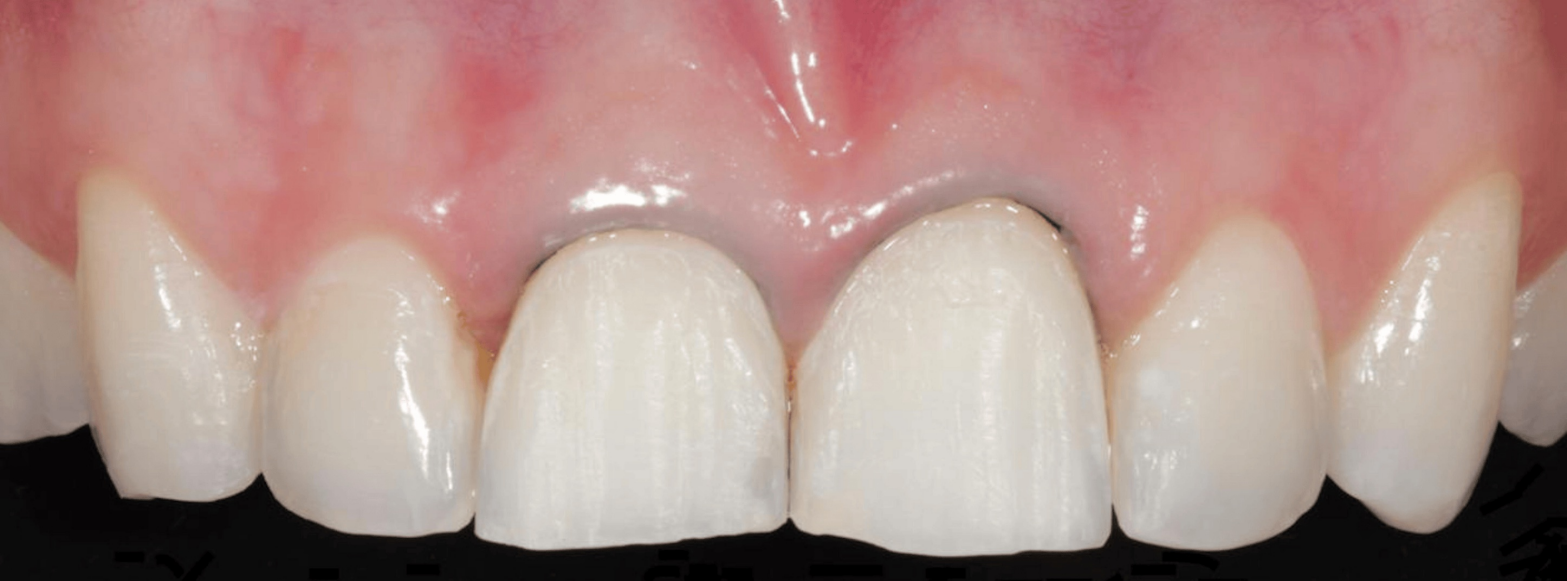
Prepared teeth ready for digital scanning

The dental preparations were done under the magnification of an Omni Zeiss Pico microscope with magnifying powers ranging from 0.4x-0.6x to 1.0x (Figure 4).

**Figure 5:**
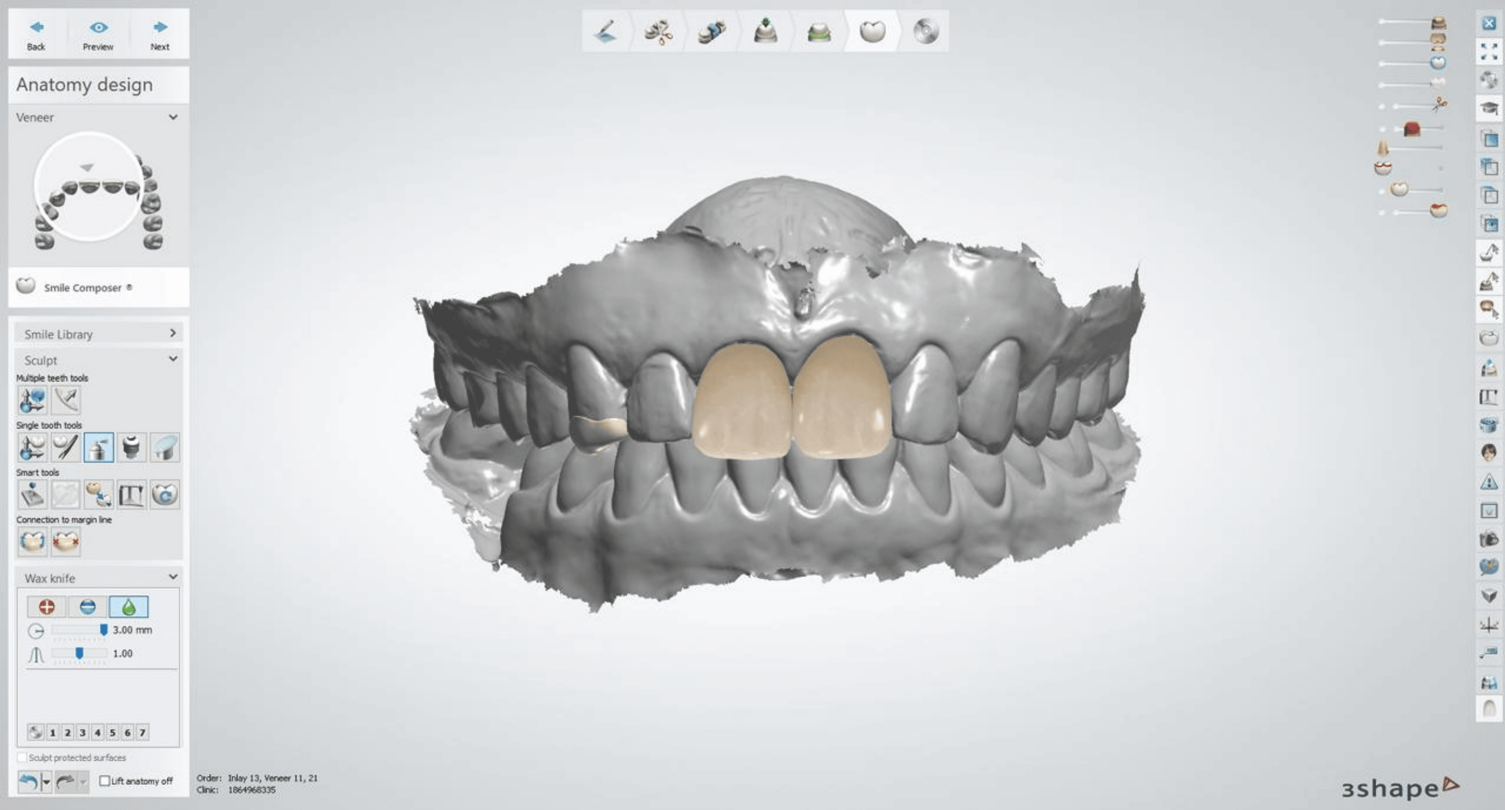
Computer-Aided Design of the restoration

After the preparations, an intraoral scan of the upper arch, lower arch, and occlusion was taken using a 3Shape Trios intraoral scanner (Figure 5) [8].

No provisionalization was needed due to the minimal nature of the preparations located in the enamel. The ceramic restorations were manufactured using a Cerec MX C machine (Dentsply Sirona, Charlotte, USA) and e.max CAD lithium disilicate material with a color of Metal Titanate (MT) A1. The material was machined at a thickness of 0.4 mm, which was in a lithium metasilicate state before crystallization. This material has a 140 megapascal (MPa) resistance, making it easier for the milling machine to work with and protect the burs. After machining, the material was crystallized to achieve its final resistance of approximately 460 MPa after sintering. Finally, the monolithic restorations were stained and glazed [9].

The ceramic restorations were tested in the patient using Choice 2 try-in paste (Bisco, Schaumburg, USA). Afterward, an adhesive cementation protocol was carried out, with relative isolation achieved using a Nic Tone dam (Nic Tone, Tokyo, Japan) [10]. The gingival sulcus was then isolated with a Zhermack 000 knitted retraction cord, and the teeth were cleaned with a prophylaxis brush and bicarbonate.

Subsequently, 9.5% Bisco hydrofluoric acid was applied for 20 seconds to condition the ceramic [11], and then the acid was neutralized and washed using bicarbonate and water for 60 seconds. After that, salt precipitates were removed using 37% phosphoric acid for one minute and rinsed thoroughly with water. A layer of bis-silane was then applied and aired on the ceramic laminates for 30 seconds, while another operator conditioned the teeth (bis-silane was prepared by combining two bottles of non-pre-hydrolyzed silane, A and B). Simultaneously, 37% Uni-etch Bisco phosphoric acid was applied for 15 seconds to the enamel of the teeth, followed by washing and drying. Afterward, All Bond Universal (Bisco, Schaumburg, USA) adhesive was applied on the same acid-treated surfaces, while rubbing, and airing until it could be observed under the microscope that the layer was not moving and remained shiny. The adhesive layer was then cured with a Bluephase lamp (Ivoclar Vivadent) for 40 seconds.

**Figure 6:**
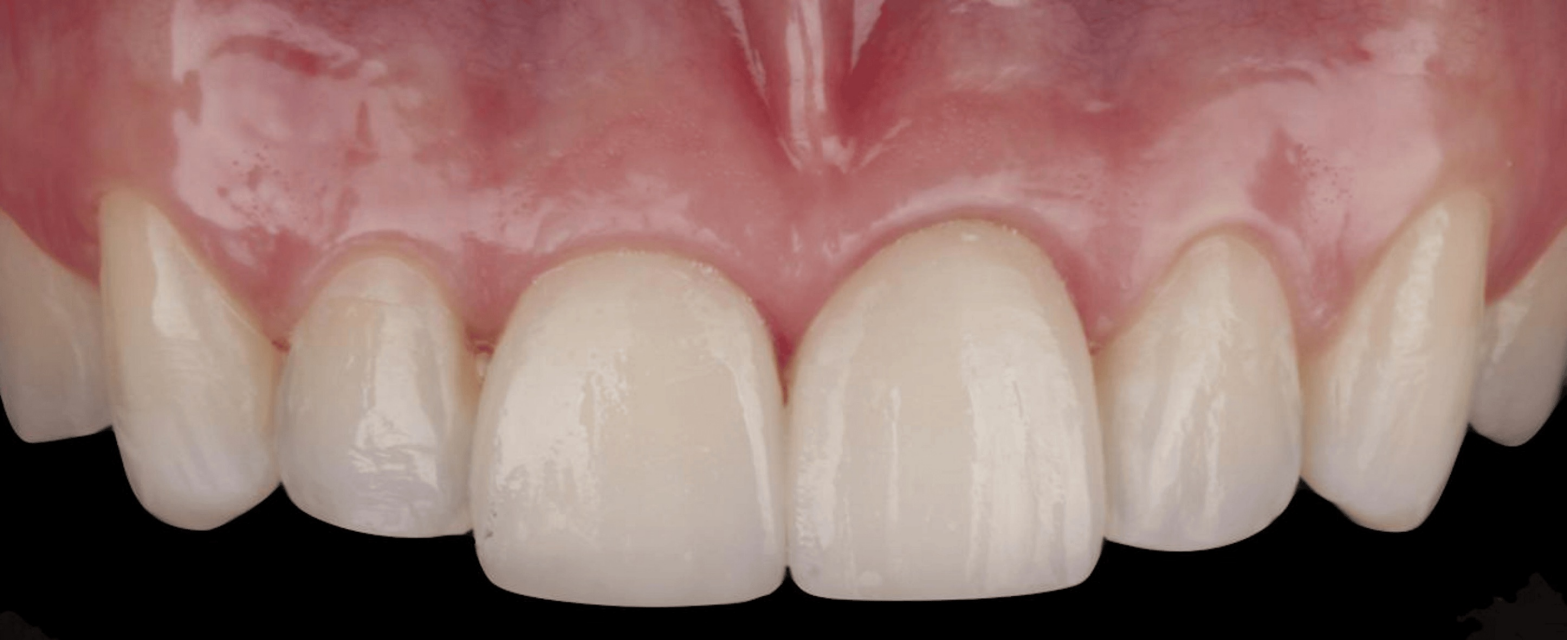
Intraoral evaluation of the completed restorations

Later, a hydroxyethyl methacrylate (HEMA) free Bisco adhesive was applied to the ceramic to promote wetting of the ceramic surface and create a hydrophobic layer that improves bonding with the cement. Next, a transparent Bisco Choice 2 light-curing cement was applied to the internal surfaces of the restoration. The excess cement was removed using brush techniques, as previously recommended by Pereira and colleagues [12] to minimize the gap between the tooth and restoration interface. The final step was curing for 40 seconds using the Bluephase lamp from 1 mm from the restoration surface, adhering to all the previously reported dental material polymerization considerations by Cadenaro et al. (Figure 6) [13].

## Discussion

The use of intraoral scanners, microscopes, and milling machines in dentistry enhances the accuracy and efficiency of Computer-Aided Design-Computer-Aided Manufacturing (CAD-CAM) restorations by allowing for precise minimal thickness analysis, preparation, and processing. According to a systematic review by Alves et al., tooth-borne ceramic restorations produced with CAD-CAM systems have a five-year success rate of 95%, comparable to those made conventionally [14].

This report highlights the benefits of a modern digital dental workflow for minimal thickness restorations. By integrating capture systems, CAD-CAM, and microscope magnification, this workflow ensures optimal results in the evaluation, preparation, adaptation, structural integrity, and optical integration of restorations, as previously reported in the literature [5,15].

Compared to traditional methods, the digital workflow offers significant advantages in precision and efficiency. The ability to create highly accurate restorations with minimal thickness not only preserves more of the natural tooth structure but also enhances the adhesion performance and longevity of the restorations. The integration of digital tools facilitates a more streamlined process, reducing chair time and improving overall clinical efficiency.

Patient outcomes are notably improved with this approach. The precision and customization enabled by digital technologies contribute to higher patient satisfaction, as seen in the case presented. The patient was able to visualize and approve the proposed restorations through the mock-up phase, leading to a more predictable and satisfactory result.

The future of digital dentistry holds promising advancements, including improved materials, more sophisticated software algorithms, and enhanced imaging techniques. These developments will likely further refine the accuracy and efficiency of restorative procedures, expanding the possibilities for minimally invasive treatments.

However, there are limitations to consider. The initial cost of digital equipment and the learning curve associated with new technologies can be barriers for some practitioners. Additionally, while the success rate is high, long-term studies are needed to further validate the durability of these restorations in various clinical scenarios.

## Conclusions

The adoption of a digital dental workflow for minimal thickness restorations offers significant benefits in terms of precision, efficiency, and patient satisfaction. Continued advancements in digital technologies are expected to further enhance the field of restorative dentistry, making it a valuable approach for modern dental practices.

## References

[1] Coachman C, Blatz MB, Bohner L, Sesma N (2021). Dental software classification and dento-facial interdisciplinary planning platform. J Esthet Restor Dent.

[2] Vandenberghe B (2018). The digital patient - imaging science in dentistry. J Dent.

[3] Apponi R, Murri Dello Diago A, Colombini V, Melis G (2021). Direct versus indirect techniques to Menage uncomplicated crown fractures of anterior teeth following dentoalveolar trauma. Dent J (Basel).

[4] Touati R, Sailer I, Marchand L, Ducret M, Strasding M (2022). Communication tools and patient satisfaction: a scoping review. J Esthet Restor Dent.

[5] Pineda-Vásquez L, Fons-Font A, Bustos-Salvador JL, Alonso-Pérez-Barquero J, Román-Rodríguez JL (2019). Shear bond strength of debonded ceramic restorations re-cemented by means of a cleaning and retreatment protocol. J Clin Exp Dent.

[6] Lawson NC, Bansal R, Burgess JO (2016). Wear, strength, modulus and hardness of CAD/CAM restorative materials. Dent Mater.

[7] Coachman C, Gurel G, Calamita M, Morimoto S, Paolucci B, Sesma N (2014). The influence of tooth color on preparation design for laminate veneers from a minimally invasive perspective: case report. Int J Periodontics Restorative Dent.

[8] Ashraf Y, Sabet A, Hamdy A, Ebeid K (2020). Influence of preparation type and tooth geometry on the accuracy of different intraoral scanners. J Prosthodont.

[9] Seghi RR, Leyva Del Rio D (2019). Biomaterials: ceramic and adhesive technologies. Dent Clin North Am.

[10] Rocca GT, Baldrich B, Saratti CM, Delgado LM, Roig M, Daher R, Krejci I (2021). Restoration's thickness and bonding tooth substrate are determining factors in minimally invasive adhesive dentistry. J Prosthodont Res.

[11] Prochnow C, Venturini AB, Guilardi LF (2018). Hydrofluoric acid concentrations: effect on the cyclic load-to-failure of machined lithium disilicate restorations. Dent Mater.

[12] Pereira S, Anami LC, Pereira CA (2016). Bacterial colonization in the marginal region of ceramic restorations: effects of different cement removal methods and polishing. Oper Dent.

[13] Cadenaro M, Maravic T, Comba A (2019). The role of polymerization in adhesive dentistry. Dent Mater.

[14] Alves de Carvalho IF, Santos Marques TM, Araújo FM, Azevedo LF, Donato H, Correia A (2018). Clinical performance of CAD/CAM tooth-supported ceramic restorations: a systematic review. Int J Periodontics Restorative Dent.

[15] Marcondes RL, Moraes RR, Pereira J, de Carvalho MA (2023). Preheated restorative composite resin for luting ceramic laminate veneers: an optimized technique report. J Clin Exp Dent.

